# Before the Tremor: Premotor Symptoms of Parkinson’s Disease

**DOI:** 10.1289/ehp.121-A342

**Published:** 2013-12-01

**Authors:** Lindsey Konkel

**Affiliations:** Lindsey Konkel is a Worcester, MA–based journalist who reports on science, health, and the environment. She writes frequently for Environmental Health News and The Daily Climate.

Clinicians currently diagnose Parkinson’s disease (PD) by the presence of tremor and impaired movement. However, PD patients also suffer from a wide range of nonmotor symptoms, some of which may appear years before the motor signs. In this issue of *EHP*, a national group of experts reviews the current body of research on these premotor symptoms, which may offer clues to better understanding the development of PD and identifying at-risk populations.^1^

“A growing body of evidence suggests that Parkinson’s disease is more than a brain disorder. Premotor symptoms offer a unique opportunity to understand the early development of the disease,” says lead author Honglei Chen, head of the Aging and Neuroepidemiology Group at the National Institute of Environmental Health Sciences.

Although there is currently no cure for PD, which affects an estimated 1–2% of people over 60,^2^ figuring out how to spot this disease earlier in its development may give researchers an opportunity to identify more effective treatments, Chen says.

Epidemiological studies suggest that nonmotor symptoms such as hyposmia (poor sense of smell), chronic constipation, rapid eye movement sleep behavior disorder (in which sufferers appear to act out dreams in their sleep), daytime sleepiness, anxiety, and depression may occur before the appearance of motor dysfunction in PD patients. Motor symptoms typically become apparent when the substantia nigra—a portion of the brain important for movement—has lost 50% or more of its dopaminergic neurons.^1^

“The clinical signs of the disease are just the tip of the iceberg. There’s a lot going on under the surface that we don’t yet recognize,” says Andrew Feigin, a PD researcher at the Feinstein Institute for Medical Research in Manhasset, New York. Feigin was not involved in the current review.

The average age at PD diagnosis is 60, according to the National Institute of Neurological Disorders and Stroke.^3^ It’s unclear how far in advance of clinical symptoms early premotor symptoms may be recognized. Clinical studies have documented the onset of rapid eye movement sleep behavior disorder or constipation 10–20 years before PD diagnosis. Data are inconsistent for the timing of other premotor symptoms.^1^

**Figure:**
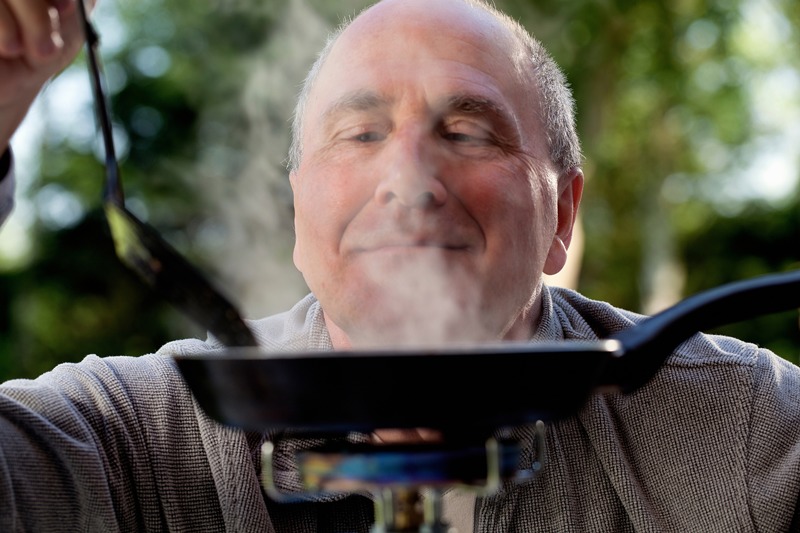
Hyposmia, or poor sense of smell, can signal Parkinson’s disease years before diagnosis. © Getty Images

Questions also remain about the usefulness of premotor symptoms in understanding the early stages of disease and identifying at-risk people. Preliminary studies suggest that a single premotor symptom itself is inadequate for predicting disease.^4^^,^^5^

Few studies have explored combinations of premotor symptoms for early disease detection, although preliminary evidence indicates that multiple symptoms may occur in people at higher risk for PD. One study found that 2 of 24 people (8.3%) with more than three premotor symptoms developed PD within 5 years of follow-up compared with just 8 of 852 people (0.9%) with one symptom.^1^

“Research on premotor symptoms is really in its infancy,” says Chen, who along with his colleagues suggests that large prospective cohort studies with lengthy follow-ups are needed to evaluate the role of premotor symptoms in PD development.

According to some experts, premotor symptoms may suggest an origin of PD that lies outside the brain.^6^^,^^7^ One hypothesis posits that abnormal protein aggregates characteristic of PD appear first in the olfactory structures and enteric nerve, then spread by a prion-like cell-to-cell transfer to the substantia nigra.^8^ Some studies suggest that PD may originate in the cells of the nasal cavity or intestines and spread to the central nervous system and the brain.^9^^,^^10^^,^^11^^,^^12^

“The idea that GI and olfactory symptoms may precede the disease by years raises the level of interest in environmental toxicants, which primarily enter the body through inhalation and ingestion,” says Jeff Bronstein, a neurologist and molecular toxicologist at the University of California, Los Angeles. Bronstein was not involved in the review.

Previous studies have reported associations between PD and certain types of pesticides^13^ or lifetime exposure to metals such as lead.^14^ Exposures to other neurotoxic chemicals, such as polychlorinated biphenyls and methylmercury, have been implicated as potential risk factors for PD, although the current body of data for these exposures remains too weak to assess.^15^
